# The *hyl*_*Efm *_gene in pHyl_Efm _of *Enterococcus faecium *is not required in pathogenesis of murine peritonitis

**DOI:** 10.1186/1471-2180-11-20

**Published:** 2011-01-25

**Authors:** Diana Panesso, Maria C Montealegre, Sandra Rincón, Maria F Mojica, Louis B Rice, Kavindra V Singh, Barbara E Murray, Cesar A Arias

**Affiliations:** 1 Department of Internal Medicine, Division of Infectious Diseases, Center for the Study of Emerging and Reemerging Pathogens, Houston, TX, USA; 2 Laboratory for Antimicrobial Research, University of Texas Medical School at Houston, Houston, TX, USA; 3 Laboratory of Enterococcal Research, University of Texas Medical School at Houston, Houston, TX, USA; 4 Department of Microbiology and Molecular Genetics, University of Texas Medical School at Houston, Houston, TX, USA; 5 Molecular Genetics and Antimicrobial Resistance Unit, Universidad El Bosque, Bogotá, Colombia; 6 Medical and Research Services, Louis Stokes Cleveland Department of Veterans Medical Center, Cleveland, OH, USA; 7 Department of Medicine, Case Western Reserve University, School of Medicine, Cleveland, OH, USA; 8 Centro Internacional de Entrenamiento e Investigaciones Médicas (CIDEIM), Cali, Colombia

## Abstract

**Background:**

Plasmids containing *hyl*_*Efm *_(pHyl_Efm_) were previously shown to increase gastrointestinal colonization and lethality of *Enterococcus faecium *in experimental peritonitis. The *hyl*_*Efm *_gene, predicting a glycosyl hydrolase, has been considered as a virulence determinant of hospital-associated *E. faecium*, although its direct contribution to virulence has not been investigated. Here, we constructed mutants of the *hyl*_*Efm*_-region and we evaluated their effect on virulence using a murine peritonitis model.

**Results:**

Five mutants of the *hyl*_*Efm*_-region of pHyl_EfmTX16 _from the sequenced endocarditis strain (TX16 [DO]) were obtained using an adaptation of the PheS* system and were evaluated in a commensal strain TX1330RF to which pHyl_EfmTX16 _was transferred by mating; these include *i*) deletion of *hyl*_*Efm *_only; *ii*) deletion of the gene downstream of *hyl*_*Efm *_(*down*) of unknown function; *iii*) deletion of *hyl*_*Efm *_plus *down*; *iv*) deletion of *hyl*_*Efm*_-*down *and two adjacent genes; and *v*) a 7,534 bp deletion including these four genes plus partial deletion of two others, with replacement by *cat*. The 7,534 bp deletion did not affect virulence of TX16 in peritonitis but, when pHyl_EfmTX16Δ7,534 _was transferred to the TX1330RF background, the transconjugant was affected in *in vitro *growth versus TX1330RF(pHyl_EfmTX16_) and was attenuated in virulence; however, neither *hyl*_*Efm *_nor *hyl*_*Efm*_-*down *restored wild type function. We did not observe any *in vivo *effect on virulence of the other deletions of the *hyl*_*Efm*_-region

**Conclusions:**

The four genes of the *hyl*_*Efm *_region (including *hyl*_*Efm*_) do not mediate the increased virulence conferred by pHyl_EfmTX16 _in murine peritonitis. The use of the markerless counterselection system PheS* should facilitate the genetic manipulation of *E. faecium *in the future.

## Background

*Enterococcus faecium *is a common enterococcal species increasingly isolated from hospital-associated infections in the USA [1]. Compelling evidence suggests that this substantial increase in *E. faecium *nosocomial infections is due to the worldwide occurrence of a genetic subcluster (designated clonal cluster 17, CC17) which encompasses clones that appear to have evolved independently [2-4]. Several genes have been associated with CC17 *E. faecium *including ***i***) *esp*_*Efm*_, encoding a surface protein which has been associated with increased biofilm formation and urinary tract infection (UTI) [4-6]; ***ii***) some *fms *genes (two of which are also designated *pilA *and *pilB*), encoding putative microbial surface components recognizing adhesive matrix molecules (MSCRAMMs) or components of enterococcal pili (including the pilus operon *ebpABC*_*fm*_, which appear to play a role in biofilm formation and experimental UTI) [2,7-10]; ***iii***) an intact *acm *gene encoding a collagen adhesin which was shown to be important in the pathogenesis of endocarditis [8] and, ***iv***) plasmids carrying the *hyl*_*Efm *_gene [11-14].

It has been previously shown that *hyl*_*Efm *_is carried by large transferable megaplasmids of different sizes (145 to 375 kb) in hospital-associated *E. faecium *which are widely distributed worldwide [11-13,15] These plasmids also can harbour antibiotic resistance determinants and some pilus-encoding genes of *E. faecium *which are present with *hyl*_*Efm *_in the same plasmid [15,16]. The acquisition of the *hyl*_*Efm*_-plasmid by an *E. faecium *laboratory strain (D344SRF) from a US clinical isolate (C68) increased the colonization of the gastrointestinal tract of mice, an effect that was independent of the presence of antibiotic resistance determinants [17]. Moreover, the acquisition of the *hyl*_*Efm*_-plasmid from another US clinical strain (TX16) increased the virulence of a commensal strain *E. faecium *TX1330RF in experimental peritonitis [11].

The Hyl_Efm _protein was initially predicted to have homology with hyaluronidases which have been associated with virulence in other gram-positive pathogens [18,19], although hyaluronidase activity has not been detected in *E. faecium *isolates carrying this gene [15]. The most recent annotation and sequence comparisons indicate that this protein is likely to encode a family 84 glycosyl hydrolase [12,13]. In fact, the homolog of *hyl*_*Efm *_in *Streptococcus pyogenes *(*spy1600*) encoded in a genetic locus with a similar organization to that of the *hyl*_*Efm*_-region and sharing 42% identity at the amino acid level (61% similarity), was recently shown not to have any detectable hyaluronidase activity. Spy1600 was characterized as a family 84 glycosyl hydrolase with β-*N*-acetyl-glucosaminidase specificity after purification and substrate analysis [20] and expression of *spy1600 *in *S. pyogenes *was found to be up-regulated during phagocytosis [21]. For this reason, and because of the almost exclusive occurrence of *hyl*_*Efm *_in isolates from clinical origin in different surveillance studies [14,22-24], this gene has been postulated as an important pathogenic determinant of hospital-associated *E. faecium*. However, its exact role in virulence has not been established. In this work, we assess the role of the *hyl*_*Efm*_-region in *E. faecium *pathogenesis of experimental peritonitis.

## Methods

### Bacterial strains and plasmids

**Figure 1:**
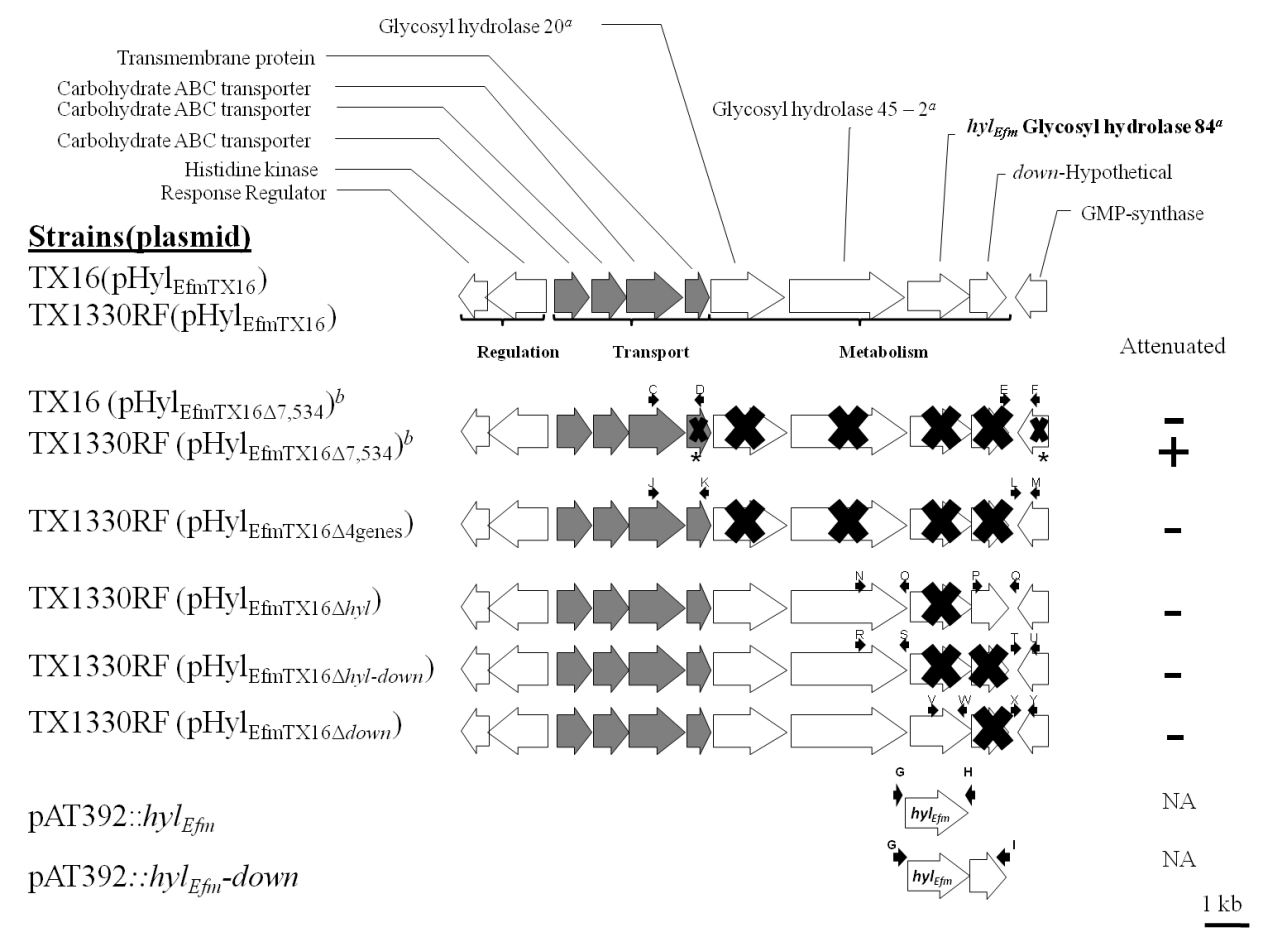
**Physical map of the *hyl***_***Efm***_**-region in pHyl**_**EfmTX16**_. The annotated predicted function of the corresponding genes is shown above the genes. The genes were divided into three groups (metabolism, transport [in gray] and regulation based on putative functions). Strain nomenclature follows that specified in Table 1. Black arrows above the genes indicate the position of the primers used to obtain DNA fragments for mutagenesis and follow the nomenclature of Table 2. The crosses depict the genes that were deleted. The asterisks indicate only partial deletion of the gene was obtained. ^*a*^The number refers to the glycosyl hydrolase family with *hyl*_*Efm *_depicted in bold; ^*b*^allelic replacement with the chloramphenicol acetyl transferase gene (*cat*) was performed. NA, not applicable.

Table 1 and Figure 1 show the strains and plasmids used in this work and depict the genetic organization of the *hyl*_*Efm*_-region in *E. faecium *strains and mutants.

**Table 1:** *E. faecium *strains and plasmids used in this work

Strains/Plasmids	Relevant Characteristics	Reference
**Strains**		
***E. faecium***		
TX16 (DO)	Sequenced endocarditis clinical isolate, Em^r^, Sm^r^. ST-16^a ^http://www.hgsc.bcm.tmc.edu	[35]
TX1330RF	Fs^r ^and Rf^r ^derivative of TX1330, a faecal colonizing strain from a healthy human volunteer	[11]
TX1330RF (pHyl_EfmTX16_)	Derivative of TX1330RF to which the *hyl*_*Efm*_-containing plasmid (pHyl_EfmTX16_) was transferred by conjugation from TX16 (DO) (~250 kb)	[11]
TX1330RF (pHyl_EfmTX16Δ7,534_)	Mutant with deletion of part or all of 6 genes of the *hyl*_*Efm *_region of TX1330RF(pHyl_EfmTX16_)	This work
TX1330RF (pHyl_EfmTX16Δ4genes_)	Non-polar deletion of 4 genes of the *hyl*_*Efm *_region of TX1330RF(pHyl_EfmTX16_)	This work
TX1330RF (pHyl_EfmTX16Δ__*hyl*_)	Non-polar deletion mutant of *hyl*_*Efm *_of TX1330RF(pHyl_EfmTX16_)	This work
TX1330RF (pHyl_EfmTX16Δ__*hyl-down*_)	Non-polar deletion of *hyl*_*Efm *_plus its downstream gene of TX1330RF(pHyl_EfmTX16_)	This work
TX1330RF (pHyl_EfmTX16Δ__*down*_)	Non-polar deletion of the gene downstream of *hyl*_*Efm *_of TX1330RF(pHyl_EfmTX16_)	This work
***E. faecalis***		
CK111	OG1Sp *upp4*::P_23 _*repA4*	[25]
**Plasmids**		
pHyl_EfmTX16_	Conjugative and transferable megaplasmid (ca. 250 kb) of TX16 (DO) containing *hyl*_*Efm*_	[11]
pCJK47	Conjugative donor plasmid for markerless mutagenesis; *oriT*_pCF10 _and pheS* pORI280 derivative; confers Em^r^	[25]
pHOU1	Derivative of pCJK47 in which the *erm*(C) gene was replaced by *aph-2'-ID*; confers Gm^r^	This work
pHOU2	Derivative of pCJK47 in which the *erm*(C) gene was replaced by *aph-2'-ID *and *cat *was incorporated in the cloning site for allelic replacements; confers Gm^r^.	This work
pTEX5501ts	*E. coli*-enterococcal shuttle plasmid for mutagenesis using a temperature-sensitive replicon	[27]
pAT392	*oriR*_pAMβ1_, *oriR*_pUC _*oriT*_RK2 _*spc lacZ*α P2 *aac(6')-aph(2")*	[30]
pAT392::*hyl*_*Efm*_	Derivative of pAT392 containing *hyl*_*Efm *_(cloned with SacI and SmaI) under the control of the P2 promoter	This work
pAT392::*hyl*_*Efm-*_*down*	Derivative of pAT392 containing both the *hyl*_*Efm *_plus downstream genes (cloned with SacI and SmaI) under the control of the P2 promoter	This work

Em^r^, erythromycin resistance; Fs^r^, fusidic acid resistance; Gm^r^, gentamicin resistance, Rf^r^, rifampin resistance; Sm^r^, high-level resistance to streptomycin. ^a^ST refers to sequence type after multi-locus sequence typing. ST16 is part of CC17

### Construction of a deletion mutant of the *hyl*_*Efm*_-region using the pheS* counter-selection system in TX16(pHyl_EfmTX16_) and its transfer to TX1330RF

**Figure 2:**
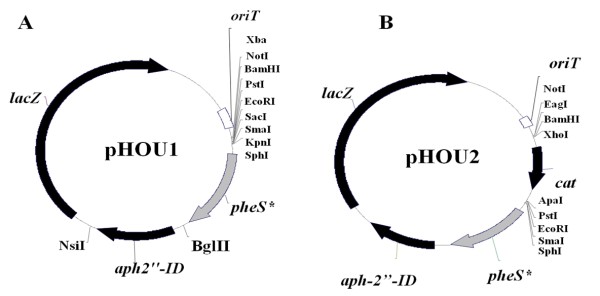
**Physical map of the plasmids pHOU1 and pHOU2 for targeted mutagenesis of *E. faecium***. A, plasmid used for construction of TX1330RF (pHyl_EfmTX16Δ4genes_), TX1330RF(pHyl_EfmTX16Δ__*hyl*_), TX1330RF(pHyl_EfmTX16Δ__*hyl-down*_) and TX1330RF (pHyl_EfmTX16Δ__*down*_) deletion mutants (Figure 1); B, plasmid used for construction of the TX1330RF(pHyl_EfmTX16Δ7,534_) deletion mutant (Figure 1)

The pheS* system (previously used in *Enterococcus faecalis*) [25] is based on the acquired sensitivity of bacteria to *p*-chloro-phenylalanine (*p*-Cl-Phe) if they carry a pheS* allele encoding a phenylalanine tRNA synthetase with altered substrate specificity [25,26]. In order to apply this approach to *E. faecium *strains, which are commonly macrolide resistant, we constructed a derivative of the pheS* vector pCJK47 by replacing its *erm*(C) gene with *aph2"-ID*, which confers resistance to gentamicin. The full *aph-2"-ID *gene (including promoter and terminator regions) was amplified by PCR using plasmid pTEX5501ts [27] as the template with primers A and B (Table 2). The amplified fragment (1,089 bp) was digested with NsiI and BglII and ligated with pCJK47 digested with the same enzymes resulting in pHOU1 (Figure 2A). Subsequently, pHOU1 was digested with BamHI and PstI and ligated with a 992 bp fragment released from pTEX5501ts after digestion with the same enzymes and containing the chloramphenicol acetyl-transferase gene (*cat*), obtaining a 7,906 bp vector designated pHOU2 (Figure 2B).

**Table 2:** Primers used in this work

Primer	Sequence (5'-3')	Relevant Characteristics
A	gaagatctgagataggttatgcaagat	Forward, BglII site (underlined), used amplification of *aph-2"-ID*
B	ccaatgcatgattccggattctaaaaaagg	Reverse, NsiI site (underlined), used amplification of *aph-2"-ID*
C	cgggatccgtttaaaaccagctggaaaag	Forward, BamHI site (underlined), located 1,251 nucleotides upstream of the start codon of the gene encoding a putative glycosyl hydrolase family 20 (Figure 1.)
D	ccgctcgagcaattcaacattgcaaagac	Reverse, XhoI site (underlined), located 294 nucleotides upstream of the start codon of the gene encoding a putative glycosyl hydrolase family 20 (Figure 1.)
E	cgagggcccgtgaagtattgccagatgt	Forward, ApaI site (underlined); located 592 nucleotides downstream of the *down *gene (hypothetical, Figure 1.)
F	ccggaattcaaaagcagaattggaaatca	Reverse, EcoRI site, 1,571 nucleotides downstream of the *down *gene (hypothetical, Figure 1.)
G	gcgagctcgattactttcaa*aggaga*	Forward, SacI site (underlined), ribosomal binding site of *hyl*_*Efm *_(italics) (Figure 1.)
H	tcccccggg*cta*acttttgataatttgctc	Reverse, SmaI site, (underlined) and stop codon of *hyl*_*Efm *_(Figure 1.)
I	tcccccggg*tta*gcgattgatcgagc	Reverse, SmaI site (underlined), stop codon of *down *(Figure 1.)
J	cgggatcccaatcaagaagtagcggatt	Forward, BamH site (underlined) 438 nucleotides upstream of the stop codon of carbohydrate ABC transporter gene (Figure 1.)
K	gcggccgctcgagggcccttagtgcgattgtatctgac	Reverse, stop codon of the gene that encodes to transmembrane protein (Figure 1.)
L	gggcccctcgaggcggccgcaaaattaaataaaaaatgg	Forward, ApaI, XhoI, NotI site, stop codon *down *(Figure 1.)
M	catgcatgaatcaggaactgaaactgc	Reverse, NsiI site, 1,091 nucleotides upstream of stop codon of GMP synthase (opposite orientation) (Figure 1.)
N	ccggaattccagtaaaaggcacagagc	Forward, EcoRI site (underlined), located 2,138 nucleotides down-stream of glycosyl hidrolase family 45-2 start codon (Figure 1.)
O	tcatctattttctcctttgaaagtaatcactatattcc	Reverse, stop codon of glycosyl hydrolase family 45-2 (Figure 1.)
P	tcaaaggagaaaatagatgaatatcttaaaaaataaaaagc	Forward, located 40 nucleotides upstream of *down *gene start codon (Figure 1.)
Q	ataagaatgcggccgcttagcgattgatcgagcg	Reverse, NotI site (underlined), stop codon of *down *(Figure 1.)
R	ataagaatgcggccgccagtaaaaggcacagagc	Forward, NotI site (underlined), located 2,138 nucleotides down-stream of glycosyl hydrolase family 45-2 start codon (Figure 1.)
S	tcatctattttctcctttgaaagtaatcactatattcc	Reverse, stop codon of glycosyl hydrolase family 45-2 (Figure 1.)
T	tcaaaggagaaaatagatgacaaaattaaataaaaaatgg	Forward, 1,973 nucleotides upstream of stop codon of GMP synthase (Figure 1.)
U	cggaattcgaatttgtatatgtcttcg	Reverse, EcoRI site (underlined), 994 nucleotides upstream of start codon of GMP synthase (opposite direction) (Figure 1.)
V	aaggaaaaaagcggccgccagaatatgataatcgtcatgg	Forward, NotI site (underlined), 902 nucleotides downstream of *hyl*_*Efm *_start codon (Figure 1.)
W	tttgttctcctttttcttgctttttattttttaag	Reverse, stop codon of of *hyl*_*Efm *_(Figure 1.)
X	gcaagaaaaaggagaacaaacaaaattaaataaaaaatgg	Forward, 1,973 nucleotides upstream of stop codon of GMP synthase (opposite direction) (Figure 1.)
Y	ccggaattcgaatcaggaactgaaactgccc	Reverse, EcoRI site (underlined), 1,094 nucleotides upstream of stop codon of GMP synthase (opposite direction) (Figure 1.)
A1	cgcgtcgtattaaaaatcat	Forward, 143 nucleotides upstream of stop codon of GH20 (Figure 3.)
A2	gatcgataaactggctcgt	Reverse, 139 nucleotides upstream of start codon of GH42 (Figure 3.)
B1	acgcgtcgacagcagctggatatgctga	Forward, SalI site (underlined), 2,316 nucleotides downstream of start codon of GH42 (Figure 3.)
B2	ggaagatctccggtttccagacttctt	Reverse, BglII site (underlined), 159 nucleotides downstream of start codon of *hyl*_*Efm *_(Figure 3.)
C1	gttagaagaagtctggaaaccg	Forward, 138 nucleotides downstream of start codon of *hyl*_*Efm *_(Figure 3.)
C2	tgctaagatattcctctactcg	Reverse, 798 nucleotides upstream of stop codon of *hyl*_*Efm *_(Figure 3.)
D1	acatgcatgcagaattggagccttggtt	Forward, SphI site (underlined), 169 nucleotides upstream of stop codon of *hyl*_*Efm *_(Figure 3.)
D2	cggaattctgcttccgcataagaaa	Reverse, EcoRI site (underlined), 319 nucleotides upstream of stop codon of *down *gene (Figure 3.)
E1	gcaaggcttcttagaga	Forward, *ddl _E. faecium _*[32,33]
E2	catcgtgtaagctaacttc	Reverse, *ddl _E. faecium _*[32,33]

In order to create a deletion mutant of the *hyl*_*Efm*_-region (which contains genes predicted to be involved in carbohydrate metabolism and transport; Figure 1), fragments upstream (977 bp) and downstream (999 bp) of this region were amplified by PCR (with primers C-D and E-F, respectively; Table 2) and cloned upstream and downstream of the *cat *gene in pHOU2, respectively, using BamHI and XhoI for the upstream fragment and ApaI and EcoRI for the downstream fragment; the correct insert was confirmed by sequencing in both directions. This recombinant plasmid was introduced into *E. faecalis *CK111 by electroporation as described previously [25,28] and blue colonies were recovered on brain heart infusion (BHI) agar plates containing gentamicin (125 μg/ml) and X-Gal (200 μg/ml). Subsequently, the pHOU2 derivatives were introduced into strain TX16 by filter mating [29] with *E. faecalis *CK111 as the donor. Single cross-over integrants were selected on gentamicin (170 μg/ml) and erythromycin (200 mg/ml) and purified colonies were then resuspended in 50 μl of normal saline and plated on MM9YEG media (salts and yeast extract) supplemented with 7 mM of *p*-Cl-Phe [25] and incubated for 48 h at 37°C. To confirm that colonies which grew on MM9YEG media supplemented with *p*-Cl-Phe were excisants, the corresponding colonies were grown simultaneously on BHI agar in the presence and absence of gentamicin. Colonies that were susceptible to gentamicin were further screened by PCR, pulsed field gel electrophoresis (PFGE) and hybridizations with *hyl*_*Efm *_and *cat *probes as described before [11]. The mutated region was also sequenced in order to confirm deletion of the corresponding genes. Subsequently, the mutated *hyl*_*Efm*_-containing plasmid (pHyl_EfmTX16Δ7,534_) was transferred from *E. faecium *TX16 to TX1330RF (a fusidic and rifampin resistant derivative of the commensal strain TX1330, Table 1) by filter mating as described previously [11] to obtain the strain TX1330RF(pHyl_EfmTX16Δ7,534_). Acquisition of the mutated plasmid by TX1330RF was also confirmed by PFGE, PCR, hybridizations and sequencing. S1 nuclease digestion and PFGE was performed with the mutant to confirm that no other plasmid had transferred during the conjugation event as previously described [11].

### Complementation of the *hyl*_*Efm*_-region mutant TX1330RF(pHyl_EfmTX16Δ7,534_)

The *hyl*_*Efm *_gene was PCR amplified with primers G and H (including the ribosomal binding site and the stop codon of *hyl*_*Efm*_) (Table 2) using total DNA from TX16 as template, and the DNA fragment (1,685 bp) cloned into the shuttle plasmid pAT392 [30] under the control of the P2 promoter (which allows constitutive expression of the cloned genes) and upstream of the *aac(6')-aph(2") *gene (which is co-transcribed from the same promoter) using SacI and SmaI sites (plasmid pAT392::*hyl*_*Efm*_). In order to evaluate if the deletion of *hyl*_*Efm *_had an effect in the downstream gene (encoding a hypothetical protein of 331 amino acids of unknown function), the *hyl*_*Efm *_and *down *genes (Figure 1) were also cloned together into pAT392 following a similar strategy and using primers G and I (pAT392::*hyl*_*Efm*_*-down*). Recombinant pAT392-derivatives were purified from *E. coli *grown on Luria-Bertani agar containing gentamicin (25 μg/ml) and all their DNA inserts sequenced. Subsequently, they were introduced into *E. faecium *TX1330RF, and the TX1330RF(pHyl_EfmTX16Δ7,534_) mutant by electroporation. Stability of the plasmid constructs was tested by isolating ca. 100 colonies from overnight cultures (using BHI broth) and from the spleens of dead animals (in different experiments) after intraperitoneal inoculation of the corresponding strain (see below) and plating them simultaneously on BHI and BHI-gentamicin (125 μg/ml).

### Construction of additional mutants of the *hyl*_*Efm*_-region in *E. faecium *TX1330RF(pHyl_EfmTX16_)

To investigate the specific role of the *hyl*_*Efm *_locus in *E. faecium *pathogenesis, complete in-frame deletions of four genes of the *hyl*_*Efm*_-region, *hyl*_*Efm *_alone, *hyl*_*Efm *_plus its downstream gene and the gene downstream of *hyl*_*Efm *_were generated using TX1330RF(pHyl_EfmTX16_). Fragments upstream and downstream of each region were amplified by PCR with the corresponding primers (Figure 1 and Table 2). These fragments, with overlapping ends, were subsequently amplified by crossover PCR and cloned into pHOU1 using EcoRI and NotI (for *hyl*_*Efm*_, *hyl*_*Efm *_plus its downstream gene and the downstream gene of *hyl*_*Efm *_mutants); and BamHI and PstI (for the four gene mutant). The inserts were sequenced in both directions to confirm that no mutations had been introduced during the cloning process. The recombinant plasmids were electroporated or transferred by conjugation (using *E. faecalis *CK111) into TX1330RF(pHyl_EfmTX16_). Single crossover events and deletions of targeted regions (Figure 1) were obtained by plating in BHI with gentamicin and *p*-Cl-Phe containing medium, respectively, as previously described [25]. Confirmation of the deletion was performed by PCR, PFGE, hybridizations and DNA sequencing.

### RT-PCR

**Figure 3:**
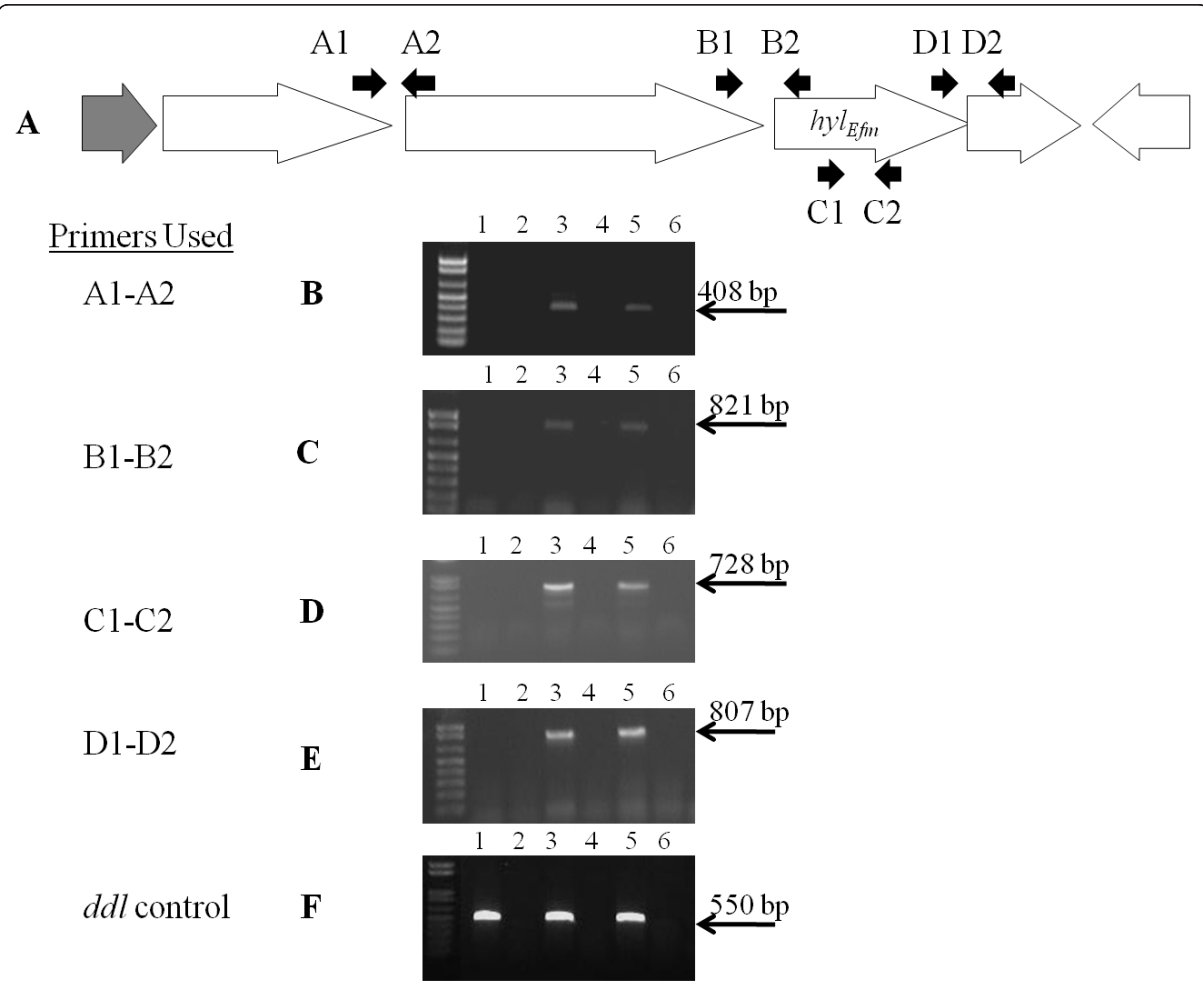
**Transcriptional analysis of genes in the *hyl***_***Efm ***_**region using reverse transcriptase (RT)-PCR**. **A**, physical map of the *hyl*_*Efm *_region and primers used for RT-PCR experiments_. _Black arrows above the genes indicate the position of the primers used to amplify DNA sequences from the cDNA obtained after reverse transcription. **B**, RT-PCR using primers A1-A2; **C**, RT-PCR using primes B1-B2; **D**, RT-PCR using primers C1-C2; **E**, RT-PCR using primers D1-D2; **F**, RT-PCR with *ddl *as the target gene using primers E1-E2 (Table 2) [32,33]. Lanes 1 and 2, TX1330RF (RT-PCR reaction and control without RT enzyme, respectively); lanes 3 and 4, TX1330RF(pHyl_Efm16_) (RT-PCR reaction and control without RT enzyme, respectively); lanes 5 and 6 TX16(pHyl_Efm16_) (RT-PCR reaction and control without RT enzyme respectively). The molecular weight of the bands is indicated to the right.

RNA was extracted from bacterial cells (TX16, TX1330RF(pHyl_EfmTX16_), TX1330RF and strains containing pAT392 derivatives) grown in BHI broth at 37°C with mild agitation (logarithmic phase of growth, *A*_600 _0.8) as described before [31], and using the RNA isolation kit RNAwiz (Ambion, Austin, TX). RNA was treated twice with DNase (DNase-Free solution, Ambion) and synthesis of cDNA was performed using the commercial kit SuperScript One-Step reverse transcription-PCR (RT-PCR) with Platinum *Taq *(Invitrogen), according to the manufacturer's instructions. The mixture contained 0.2 μM of each primer, designed to detect overlapping transcripts of the four putative metabolic genes (Figure 3) and an internal transcript of *hyl*_*Efm *_(Table 2). A primer pair directed to detect a 550-bp transcript of the housekeeping gene *ddl*_*E. faecium *_was used as an internal control for RT-PCR experiments [32,33].

### Mouse peritonitis model

Female (4 to 6 week old), outbred ICR mice (Harlan Sprague Dawley, Houston) were used as previously described [34]. Groups of 10 mice per inoculum (ranging from 2.3 × 10^8 ^to 3.1 × 10^9 ^CFU/ml) were included in each experiment. Inocula for each peritonitis experiment were prepared by growing bacteria initially on BHI agar plates. Subsequently, one colony was grown in BHI broth for 24 h at 37°C and the cells were concentrated in saline (0.9%) to an *A*_600 _of ca. 1.2. Strains containing pAT392 and derivatives were handled similarly before the intraperitoneal inoculation, except that the BHI agar and broth contained gentamicin (125 μg/ml). Comparison of the survival curves at similar inocula was performed using a log-rank test with Prism for Windows^®^. A *P *< 0.05 was considered significant. All experiments were approved by the Animal Welfare committee, University of Texas Health Science Center at Houston.

## Results and Discussion

### Deletion of 6 genes in the *E. faecium hyl*_*Efm*_-region altered *in vitro *growth and attenuated virulence of TX1330RF(pHyl_EfmTX16_) but not TX16(pHyl_EfmTX16_) in murine peritonitis

**Figure 4:**
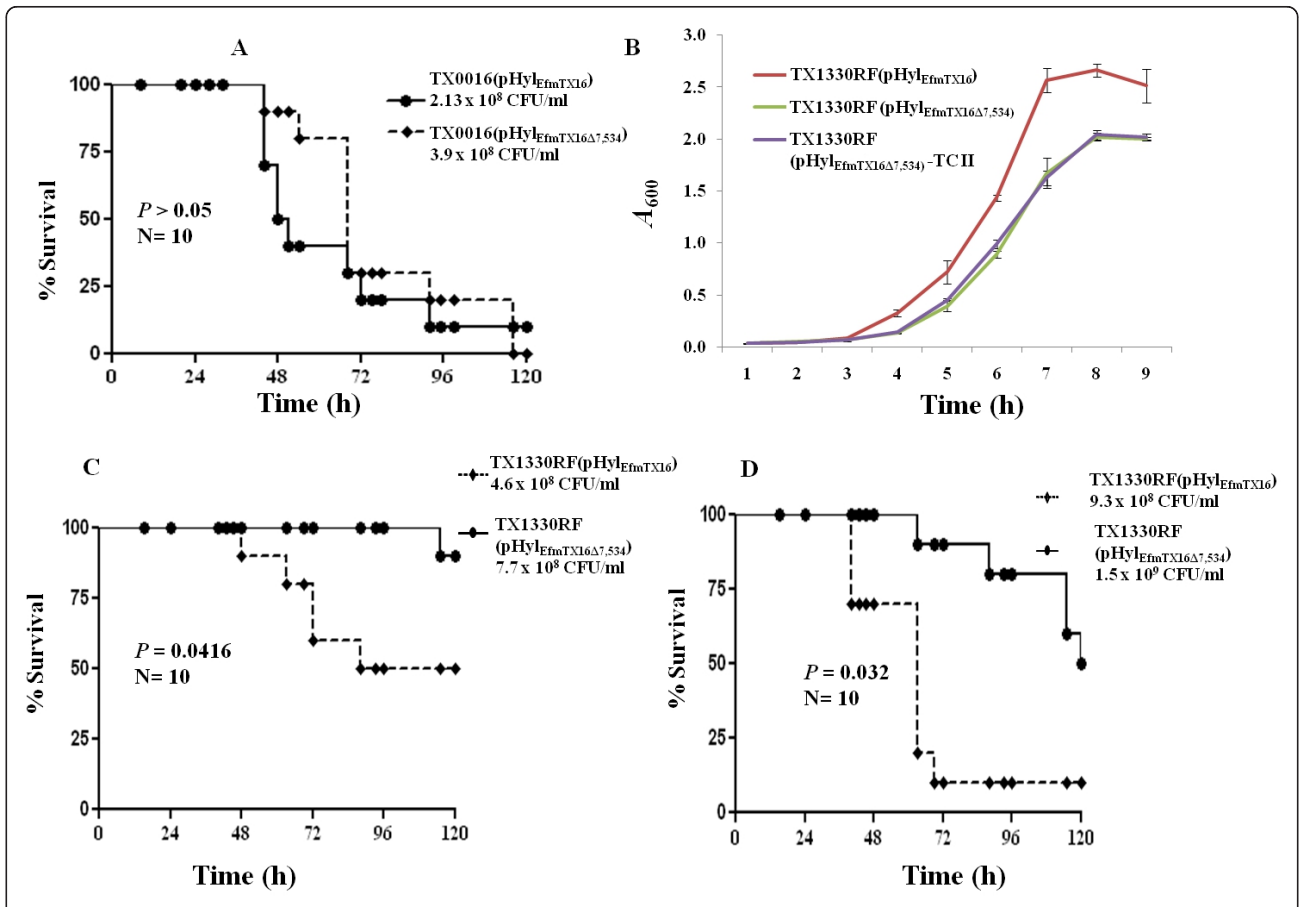
**Growth and survival curves in the mouse peritonitis model of *E. faecium *TX0016(pHyl**_**EfmTX16**_**) and TX1330RF(pHyl**_**EfmTX16**_**), carrying an intact *hyl***_***Efm***_**-region, and pHyl**_**EfmTX16Δ7,534 **_**(6 gene mutant of the *hyl***_***Efm***_**-region)**. **A**, Survival curve of representative inoculum (5 inocula per experiment in two independent experiments) of TX0016(pHyl_EfmTX16_) vs TX0016(pHyl_EfmTX16Δ7,534_) in mouse peritonitis; **B**, growth curves of TX1330RF(pHyl_EfmTX16_) vs TX1330RF(pHyl_EfmTX16Δ7,534_) and a second transconjugant [TX1330RF(pHyl_EfmTX16Δ7,534_)-TCII] obtained from the same mating experiment between TX16(pHyl_EfmTX16Δ7,534_) and TX1330RF, expressed as optical density (*A*_600_) in brain heart infusion (BHI) broth (results of at least three experiments per strain). **C **and **D**, survival curves of TX1330RF(pHyl_EfmTX16_) vs TX1330RF(pHyl_EfmTX16Δ7,534_) obtained in the peritonitis model at different inocula in independent experiments performed at different days.

Since acquisition of the transferable pHyl_EfmTX16 _by TX1330RF conferred increased virulence in experimental peritonitis [11], we explored the possibility that the *hyl*_*Efm *_region was an important mediator of this effect. Using RT-PCR assays, we were able to detect *in vitro *expression of *hyl*_*Efm *_during the exponential phase of growth in both TX16 and TX1330RF (pHyl_EfmTX16_) (Figure 3). RT-PCR with primers located at the 3' and 5' ends of contiguous genes yielded products of the expected size in each case, suggesting that these genes are likely to be co-transcribed (Figure 3). Then, we adapted the pheS* counter-selection system [25] developed for *E. faecalis *to obtain several deletions of the *hyl*_*Efm*_-region. The *hyl*_*Efm *_gene in *E. faecium *TX16 (http://www.ncbi.nlm.nih.gov/genomeprj/30627, Genbank accession number ACIY00000000) is located in a cluster of genes whose putative function appears to involve the transport and breakdown of carbohydrates (Figure 1) [13]. As an initial step to test the mutagenesis system, a relatively large deletion (7,534 bp) from pHyl_EfmTX16 _was obtained. The deletion involved three genes predicted to encode glycosyl hydrolases (including *hyl*_*Efm*_) and a gene downstream of *hyl*_*Efm *_whose function is unknown (Figure 1). Part (226 nucleotides) of a gene encoding a hypothetical transmembrane protein and located upstream of the putative family 20 glycosyl hydrolase gene and part (202 nucleotides) of a gene located 1,332 nt downstream of *hyl*_*Efm *_encoding a putative GMP-synthase and likely transcribed in the opposite direction from the *hyl*_*Efm *_cluster (Figure 1) were also deleted. As it is shown in Figure 4A, the deletion of 7,534 bp in the *hyl*_*Efm*_-region did not affect the virulence of TX16 (DO) in murine peritonitis.

Next, we considered the possibility that an *in vivo *effect might be more clearly dissected if studies were performed in the background of a non-clinical strain. We hypothesized that an *in vivo *effect of a virulence determinant might more likely be seen in strains which are less successful clinically; that is, that a commensal strain such as TX1330RF [11] is likely to have decreased fitness or ability to produce disease compared to TX16 [35] and, thus, acquisition plus subsequent loss of a virulence determinant that alters such fitness would be easier to identify [11]. Thus, the mutated plasmid from strain TX16(pHyl_EfmTX16Δ7,534_) was transferred to TX1330RF by conjugation and the *in vivo *effect of acquiring the intact plasmid [11] vs the plasmid carrying the deletion was evaluated. The two strains [TX1330RF(pHyl_EfmTX16_) and TX1330RF(pHyl_EfmTX16Δ7,534_)] appeared to differ only in the size of the *hyl*_*Efm *_plasmid by PFGE and S1 nuclease assays [11] (not shown). Figure 4B shows that deletion of 7,534 bp in the *hyl*_*Efm *_region of TX1330RF(pHyl_EfmTX16_) caused an *in vitro *growth defect. The alteration of growth was also seen in a second transconjugant from the same mating experiment between TX16(pHyl_EfmTX16Δ7,534_) and TX1330RF (TC-II in Figure 4B). The mutant strain TX1330RF(pHyl_EfmTX16Δ7,534_) was attenuated in the mouse model of peritonitis (even when an increased intraperitoneal inoculum for the mutant were used) (Figure 4C and 4D) (P < 0.05). Due to the alterations produced in the growth of TX1330RF(pHyl_EfmTX16Δ7,534_), these results suggest that the attenuation in virulence may have also been due to factors other than those specifically related to virulence.

### Complementation of the *hyl*_*Efm*_-region mutant with *hyl*_*Efm *_and a combination of *hyl*_*Efm *_and the downstream gene did not restore the virulence of TX1330RF(pHyl_EfmTX16Δ7,534_)

**Figure 5:**
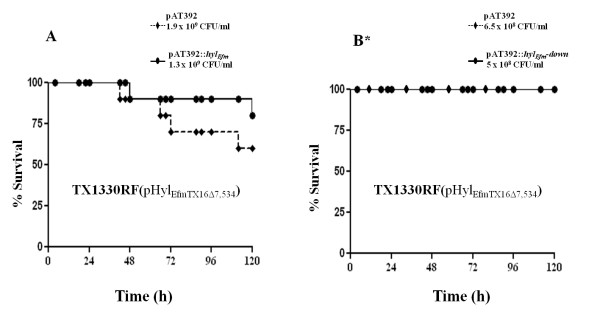
**Survival curves in the mouse peritonitis model of *E. faecium *TX1330RF and derivatives**. **A **and **B **show survival curves of the TX1330RF(pHyl_EfmTX16Δ7,534_) (6 gene mutant in the *hyl*_*Efm *_region) complemented with pAT392-derivatives (which include pAT392::*hyl*_*Efm *_and pAT392::*hyl*_*Efm*_*-down*) obtained in the peritonitis model at different inocula in independent experiments performed at different days. The asterisk indicates that the lines are superimposed since values are identical.

In order to further evaluate if the attenuation observed in TX1330RF(pHyl_EfmTX16Δ7,534_) (as described above) was mediated by a direct effect of *hyl*_*Efm *_in the peritonitis model, we explored complementation of this mutant *in trans *with the full *hyl*_*Efm *_gene and a combination of *hyl*_*Efm *_and the downstream gene using the shuttle vector pAT392 [30]. The cloning strategy placed these genes upstream of the *aac(6')-aph(2") *gene (which confers resistance to gentamicin) resulting in all open reading frames under the control of the constitutive P2 promoter. Up to 80% loss was observed with all strains in the absence of gentamicin; however, in the presence of the antibiotic during inoculum preparation, the TX1330RF(pHyl_EfmTX16Δ7,534_)-derivatives containing the pAT392 constructs were stable both *in vitro *and *in vivo *(5% maximum percentage of plasmid loss). Introduction of *hyl*_*Efm *_or a combination of *hyl*_*Efm *_plus its downstream gene (cloned into pAT392) did not restore the virulence of the mutant strain TX1330RF(pHyl_EfmTX16Δ7,534_), compared to pAT392 alone in the presence of gentamicin (Figure 5A and 5B). The results indicate that constitutive expression of *hyl*_*Efm *_alone or in combination with its downstream gene (which was confirmed by RT-PCR, not shown) was not able to restore the phenotypic differences observed in the mutant strain TX1330RF(pHyl_EfmTX16Δ7,534_), supporting the fact that *hyl*_*Efm *_may not be directly responsible of the attenuation observed in the mutant.

Under our experimental conditions, we cannot completely rule out that the *in vivo *attenuation observed with pHyl_EfmTX16Δ7,534 _in the TX1330RF background may have been caused by the partial deletion of the hypothetical transmembrane protein or the putative GMP-synthase located upstream and downstream of the *hyl*_*Efm*_-cluster, respectively. Indeed, a deletion of 76 amino acids in the C-terminus of the hypothetical membrane protein occurred in this plasmid, resulting in the deletion of three predicted transmembrane helices. Similarly, 68 amino acids in the C-terminus of the putative GMP-synthase were deleted; the removal of these amino acids is likely to disturb the dimerization domain of this protein [36] affecting its function in nucleotide metabolism. Moreover, a second TX1330RF(pHyl_EfmTX16Δ7,534_) mutant also exhibited an almost identical growth defect (Figure 4B). Thus, it is tempting to speculate that changes in these two genes may have affected the "metabolic" fitness of the TX1330RF(pHyl_EfmTX16Δ7,534_) strain. However, since no evident change in fitness or virulence was observed with the mutated plasmid in the TX16 background, another possibility is that an extraneous change elsewhere in the plasmid (or chromosome) occurred during the conjugation process that influenced the *in vitro *growth of the TX1330RF(pHyl_EfmTX16Δ7,534_) mutant(s) and its virulence.

### Additional deletions of genes in the *hyl*_*Efm*_-region did not alter the virulence of TX1330RF(pHyl_EfmTX16_) in the mouse peritonitis model

**Figure 6:**
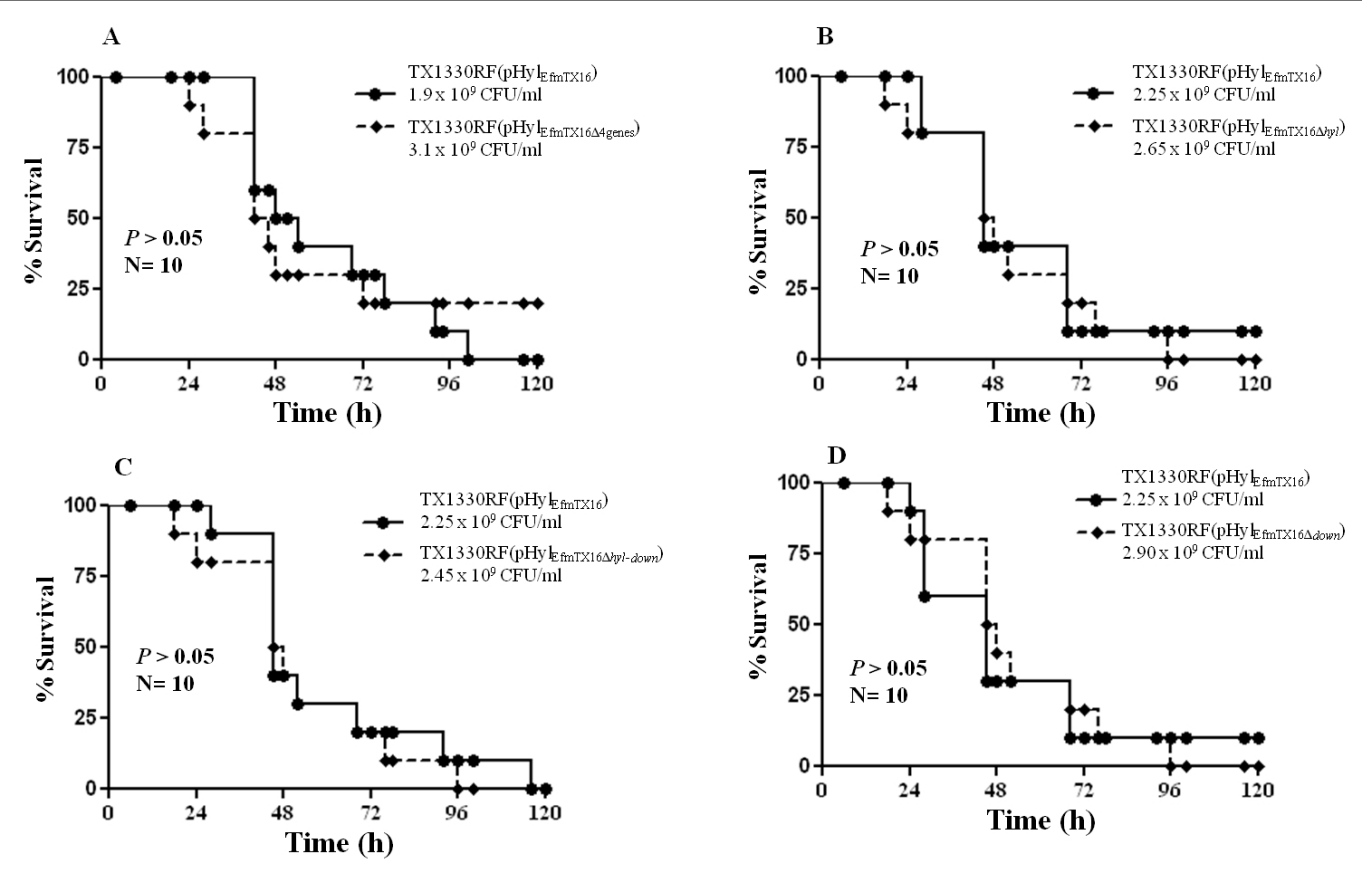
**Survival curves in the mouse peritonitis model of *E. faecium *TX1330RF(pHyl**_**EfmTX16**_**) and deletion mutants (Figure 1 and Table 1) showing representative inocula (5 inocula per each experiment)**. **A**, TX1330RF(pHyl_EfmTX16_) vs TX1330RF(pHyl_EfmTX16Δ4genes_); **B**, TX1330RF(pHyl_EfmTX16_) vs TX1330RF (pHyl_EfmTX16Δhyl_); **C**, TX1330RF(pHyl_EfmTX16_) vs TX1330RF(pHyl_EfmTX16Δ__*hyl-down*_); **D**, TX1330RF(pHyl_EfmTX16_) vs TX1330RF(pHyl_EfmTX16Δ__*down*_)

In order to dissect further the *in vivo *role of *hyl*_*Efm *_and the adjacent genes, we produced several in-frame deletions of these genes (Figure 1) including: ***i***) a four gene mutant of the *hyl*_*Efm*_-region (including *hyl*_*Efm*_) [TX1330RF(pHyl_EfmTX16Δ4genes_)], ***ii***) a deletion of *hyl*_*Efm *_alone [TX1330RF (pHyl_EfmTX16Δ__*hyl*_)], ***iii***) a deletion of *hyl*_*Efm *_plus its downstream gene mutant [TX1330RF (pHyl_EfmTX16Δ__*hyl-down*_)] and, ***iv***) a single deletion of the gene located downstream from *hyl*_*Efm *_[TX1330RF (pHyl_EfmTX16Δ__*down*_)]. The mutagenesis strategy removed the open reading frame from the start codon of the first gene to the stop codon of the last gene (in case of multiple genes). In case of single gene deletion, the complete ORF (start to stop codon) was removed, leaving the surrounding DNA intact as in the wild type plasmid. None of the four mutants of the *hyl*_*Efm*_-region showed a deleterious effect in the growth kinetics compared to TX1330RF (pHyl_EfmTX16_) (harbouring an intact plasmid, Additional file 1). Moreover, we were unable to observe any attenuation of virulence in the mouse peritonitis model compared to the parental strain with the intact plasmid (Figure 6A-D), which further supports the fact that the four genes of the *hyl*_*Efm *_region do not appear to be directly involved in increasing the pathogenic potential of pHyl_EfmTX16 _in strain TX1330RF(pHyl_EfmTX16_).

Megaplasmids (>145 kb, with or without *hyl*_*Efm*_) have been recently found to be widespread among clinical isolates of *E. faecium *worldwide [12,13,15]. The proportion of these plasmids carrying *hyl*_*Efm *_appears to vary according to geographical location (ca. 11 to 36%) [12,13]. Our findings indicate that the four genes of the *hyl*_*Efm*_-cluster studied here, including *hyl*_*Efm *_are not the main mediators of the virulence effect conferred by the plasmid carrying them in experimental peritonitis. Since the pHyl_Efm _plasmids are large, it is presumed that other genes (i.e., upstream or downstream of the glycoside hydrolase-encoding genes) are more relevant in mediating this effect. Additionally, we cannot exclude that the *hyl*_*Efm *_cluster studied in this work may play a role in other infections such as endocarditis or urinary tract infections (a subject of our ongoing studies). As a final remark, the adaptation of the pheS* counter-selection system for targeted mutagenesis in plasmid and chromosomal genes of *E. faecium *will facilitate the understanding of the role of other specific plasmid genes in the pathogenesis of *E. faecium *infections in the near future.

## Conclusions

We provided evidence that four genes of the *hyl*_*Efm*_-region (including *hyl*_*Efm*_) do not mediate the virulence effect of the *E. faecium *plasmid pHyl_Efm _in experimental peritonitis. The adaptation of the PheS* counter-selection system for targeted mutagenesis of *E. faecium *should facilitate the study of the role of other pHyl_Efm _genes in the pathogenesis of murine peritonitis.

## Competing interests

The authors declare that they have no competing interests.

## Authors' contributions

DP carried out molecular genetics studies, animal experiments and participated in editing the manuscript. MCM, SR and MFM performed molecular genetics experiments. KVS carried out part of the animal work. BEM and LBR participated in editing the manuscript and data analysis. CAA is the principal investigator, conceived the study, designed the experiments, performed data analysis and wrote the manuscript. All authors read and approved the final version of the manuscript.

## Supplementary Material

Additional file 1 **Growth curves of *E. faecium *and mutants**. The strains were incubated in BHI broth and the *A*_600 _were measured every hour. Click here for file
