# Supplementary figures and images for: The hylEfm gene in pHylEfm of Enterococcus faecium is not required in pathogenesis of murine peritonitis
Source: BMC Microbiol. 2011 Jan 25;11:20. doi: 10.1186/1471-2180-11-20 (PMC3039558; doi:10.1186/1471-2180-11-20)

## Slide 1
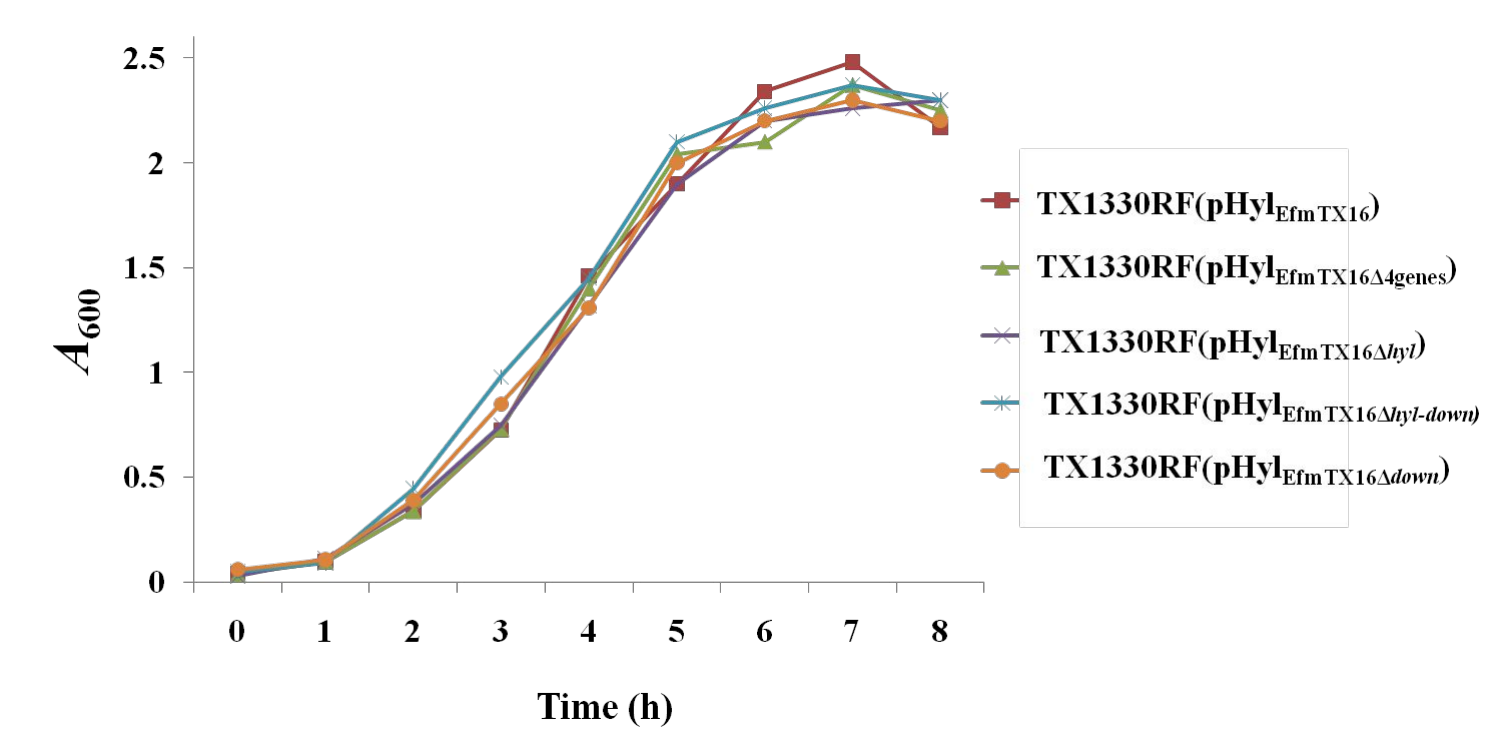

Supplement: Additional file 1 — Growth curves of E. faecium and mutants. The strains were incubated in BHI broth and the A600 were measured every hour. [file 1471-2180-11-20-S1.PPTX]
